# Unveiling the Dietary Selection of Lowland Tapirs (*Tapirus terrestris*) in a Tropical Rainforest

**DOI:** 10.1002/ece3.73161

**Published:** 2026-02-25

**Authors:** Laís Lautenschlager, Yuri Souza, Luísa Genes, Bruno H. Saranholi, Carla C. Cristina Gestich, Carina I. Motta, Valesca B. Zipparro, Pedro Galetti, Mauro Galetti, Kenneth J. Feeley

**Affiliations:** ^1^ Department of Biology University of Miami Coral Gables Florida USA; ^2^ Iniciativa Nacional para a Conservação da Anta Brasileira (INCAB) / Lowland Tapir Conservation Initiative (LTCI) Instituto de Pesquisas Ecológicas (IPÊ) Campo Grande MS Brazil; ^3^ Department of Biology Stanford University Stanford California USA; ^4^ Refauna Rio de Janeiro RJ Brazil; ^5^ Departamento de Genética e Evolução Universidade Federal de São Carlos (UFSCar) São Carlos SP Brazil; ^6^ Instituto Tecnológico Vale Belém PA Brazil; ^7^ Departamento de Biodiversidade, Centro de Pesquisa em Biodiversidade e Mudanças do Clima (CBioClima) Universidade Estadual Paulista (UNESP) Rio Claro SP Brazil

**Keywords:** Atlantic forest, conservation, feeding ecology, large mammalian herbivores, molecular diet, plant functional traits

## Abstract

Large terrestrial herbivores play crucial roles in shaping ecosystem structure and function through their foraging activities. Still, the dietary ecology of elusive tropical species remains poorly understood. We investigated the diet composition of lowland tapirs ( *Tapirus terrestris* ), the largest terrestrial herbivore in the Neotropics, using DNA metabarcoding of fecal samples from 31 latrines in Carlos Botelho State Park, Brazil. We characterized local plant communities through vegetation plots and analyzed five leaf economic spectrum (LES) traits from both consumed and surrounding vegetation to assess selective feeding patterns. Lowland tapirs consumed 61 plant species from 69 genera and 46 families, predominantly those from the Melastomataceae, Asteraceae, and Myrtaceae families. Beta‐diversity analysis revealed high compositional turnover among latrines, with a high dissimilarity index, indicating that the samples being compared are distinct in species composition. The plant composition in tapir diets differed significantly from that of the surrounding vegetation, suggesting that this species forages on distinct plant species across its extensive home range rather than consuming locally abundant species. Finally, the functional trait analysis revealed no significant differences between the dietary species and the surrounding vegetation in LES traits. Tapirs consumed plants that spanned both “fast” (high specific leaf area and high nitrogen content) and “slow” (high leaf dry matter content and thick leaves) strategies, indicating a broad dietary tolerance rather than trait‐based selectivity. This suggests that tapirs can adapt to diverse plant textures and nutritional profiles, browsing on leaves ranging from tough to softer and more digestible. Our findings demonstrate that lowland tapirs exhibit generalist feeding strategies, which promote high plant species turnover, potentially contributing to the maintenance of tropical forest diversity, as observed in the Atlantic forest. Given the critical threats facing this endangered megafauna, understanding their generalist diet is essential for developing effective conservation strategies.

## Graphical abstract

Large terrestrial herbivores, such as lowland tapirs (*Tapirus terrestris*) present many dietary ecological roles in their habitat. In this article, we investigated the diet of lowland tapirs in a tropical rainforest combining different techniques such as DNA metabarcoding, fieldwork, and plant functional traits. Our results suggests that this large herbivore is a generalist feeder, consuming 61 plant species, enhancing diversity turnover within latrines, and not consuming the immediate surrounding vegetation. We also found that this species consume plants with both ends of the leaf economic spectrum traits. We discuss the importance of tapirs for the plant biodiversity and conservation of this endangered animal.

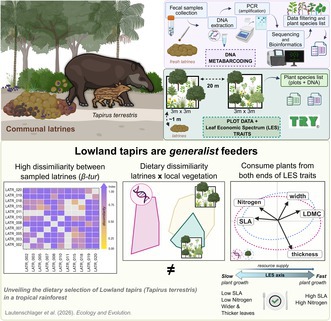

## 1 Introduction

Large terrestrial herbivores (> 10 kg body mass; Sandom et al. 2014) are known to shape ecosystem structure and function through complex trophic and non‐trophic mechanisms (Dirzo et al. 2014; Forbes et al. 2019). These megafaunal species act as critical regulators of plant community composition and dynamics, preventing plant hyperdominance by a few plant species (Souza et al. 2022) and thereby contributing to the formation and structure of diverse tropical forests. Their ecological role extends beyond herbivory, with experimental studies revealing cascading effects on beta‐diversity patterns through nutrient cycling and biogeochemical flows across multiple spatial scales (Villar et al. 2020, 2021). Furthermore, large herbivores can enhance ecosystem resilience by promoting soil carbon sequestration and stability, thereby providing a crucial buffering capacity against the impacts of climate change and other global environmental pressures (Villar and Medici 2021; Kristensen et al. 2022).

Understanding the foraging ecologies of large herbivores is crucial for comprehending their diversified plant–animal interactions, including both antagonistic effects (e.g., herbivory, seed predation, or trampling pressures on vegetation) and mutualistic relationships (e.g., seed dispersal and nutrient cycling) (Forbes et al. 2019). The impacts of large herbivores on terrestrial ecosystems vary, as the amount and type of phytomass consumed depend on differences in density, activity, and dietary patterns among the animals (Pringle et al. 2023). For instance, in African savannas, megaherbivores such as the greater kudu ( *Tragelaphus strepsiceros* ), sables ( *Hippotragus niger* ), zebras (*Equus* spp.), and even elephants ( *Loxodonta africana* ) comprise a wide range of dietary selections and feeding habits from grazers, browsers, and mixed‐feeders, with well‐studied food plant partitioning in space and time (Kartzinel and Pringle 2020; Potter et al. 2022).

In Neotropical forests, large herbivores include species such as peccaries (*Dicotyles tajacu* and *Tayassu pecari* ), capybaras ( *Hydrochoerus hydrochaeris* ), several deer species (e.g., *Blastocerus dichotomus* , *Ozotoceros bezoarticus* , and *Mazama* spp.), and the lowland tapir, *Tapirus terrestris* . Tapirs are recognized as one of the largest terrestrial mammals in the Neotropics, with an adult weight range from 200 to 250 kg (Giombini et al. 2009). Its geographic distribution spans low altitudes of northern and central South America, including tropical rainforests, flooded grasslands, and savannas (Rivera et al. 2021). This species consumes a wide variety of leaves, fruits, and aquatic vegetation, playing important roles in tropical ecosystem dynamics, such as being browsers, seed dispersers, and seed predators (Naranjo 2019; O'Farrill et al. 2013). Lowland tapirs exhibit dietary differences in response to varying biomes, habitats, and resource availability (Galetti et al. 2001; Fragoso et al. 2003; Hannibal et al. 2019). For instance, in Brazilian savannas, their diet is primarily composed of browsing on leaves and stems, with an increase in fruit consumption during the dry season (Talamoni and Assis 2009). In Amazon forests, tapirs' diets are mostly influenced by seasonal variations, with frugivory habits peaking during fruit‐rich seasons, while consumption of leaves and other vegetative parts occurs during leaner months (Vélez et al. 2017). This broad diet enables lowland tapirs to exploit various plant materials according to availability, thereby influencing the structure of plant communities (Naranjo 2019). Tapirs frequently defecate in communal latrines—sites where multiple individuals repeatedly defecate, often shared among members of the same family, social group, or neighboring groups (Buesching and Jordan 2019; Lautenschlager et al. 2024). Because these latrines integrate the fecal material of several individuals over time, they provide a unique, collective record of dietary intake. Thus, analyzing samples from communal latrines offers a powerful proxy for understanding the population‐level diet of lowland tapirs, rather than the diet of isolated individuals (Acosta et al. 1996).

The elusive nature of forest‐dwelling tropical large herbivores, including lowland tapirs, often requires indirect methodological approaches for investigating foraging ecology and diet. Traditional methods include examining browsing signs (Salas and Fuller 1996), as well as macroscopic and microscopic analyses of the stomach (Bodmer 1990, 1991) and fecal content analysis (Tobler et al. 2010; O'Farrill et al. 2013). However, these approaches are limited by low resolution in identifying consumed species, which are sometimes restricted to plant families, providing an incomplete characterization of dietary patterns and resource selection mechanisms, especially in highly diverse habitats such as tropical forests.

Recent advances in molecular techniques have transformed herbivore dietary studies, particularly through DNA metabarcoding approaches in African savannas and temperate ecosystems (Pompanon et al. 2012; Kartzinel et al. 2015; Deagle et al. 2019; Kartzinel and Pringle 2020), with only a few recent studies in neotropical ecosystems (Hibert et al. 2013; Mata et al. 2024; Martinelli Marín et al. 2024; Barreto et al. 2025). DNA metabarcoding enables species identification from fecal samples by extracting, amplifying, and sequencing specific molecular markers (Pompanon et al. 2012; Ando et al. 2020), which reveal cryptic dietary variation through the identification of plant material in fecal samples (Pansu et al. 2019, 2022). Additionally, DNA metabarcoding has demonstrated high sensitivity, fine taxonomic resolution, and considerable cost‐effectiveness (Ando et al. 2020).

A comprehensive understanding of herbivore diet ecology requires integration of a detailed taxonomic identification with functional trait analysis, so that we can elucidate the mechanisms underlying resource selection. Plant functional traits have been well‐studied, as they mediate community dynamics (McGill et al. 2006; Kraft et al. 2015), with leaf traits being particularly relevant for large herbivores that consume substantial quantities of foliage (Owen‐Smith 1982). One useful framework for analyzing these leaf traits in the context of herbivore nutrition is the leaf economic spectrum (LES). The LES framework describes general trade‐offs in leaf functional characteristics: species at the “fast” end of the LES invest heavily in metabolically active cell contents that promote rapid growth while minimizing structural investments and leaf longevity, whereas “slow” species exhibit opposite allocation patterns (Wright et al. 2004; Reich 2014; Díaz et al. 2022). For instance, fast plants typically exhibit traits such as high specific leaf area (SLA) and nitrogen content, which support rapid photosynthesis and growth (Evans 1989; Poorter et al. 2009). In contrast, slow species typically exhibit high leaf dry matter content (LDMC) and thick leaves featuring heavily lignified cell walls, which reflect greater structural investment (Onoda et al. 2017; Osnas et al. 2013; Wright et al. 2004) and greater leaf longevity. Since cell contents are more digestible than cell walls for animals (Van Soest 1994), the balance between cell wall and cell content investment affects plant palatability, leading herbivores to favor fast plants on the LES.

The dietary preferences and underlying functional drivers of large herbivores are poorly understood, particularly for cryptic species like tapirs in tropical rainforests. To fill this knowledge gap, we integrated DNA metabarcoding with plant functional trait analysis to investigate the feeding ecology of lowland tapirs. This combined approach offers novel insights into resource selection, thereby enhancing our understanding of plant‐herbivore interactions in hyperdiverse tropical ecosystems. Here, we aimed to investigate (1) which plant species are consumed by tapirs, (2) whether the composition of consumed plant species differs among different latrines sampled, (3) how the species they consume differ from the local flora composition, and (4) how the LES traits of consumed plant species compare to those of the local flora in a tropical rainforest. Given the extensive home ranges and high mobility of lowland tapirs (mean movement speed of 11.2 km/day, Medici et al. 2022), we hypothesized that tapirs would exhibit generalist dietary patterns characterized by substantial variation in species composition among latrines, resulting in high turnover of consumed plant species. However, within this generalist framework, we predicted that tapirs would demonstrate selective feeding based on LES associated with differences in palatability and nutritional quality. Specifically, we expected tapirs to preferentially consume plants positioned at the fast end of the LES, characterized by high SLA, low LDMC, and elevated nitrogen concentrations.

## 2 Materials and Methods

### 2.1 Study Site

**FIGURE 1:**
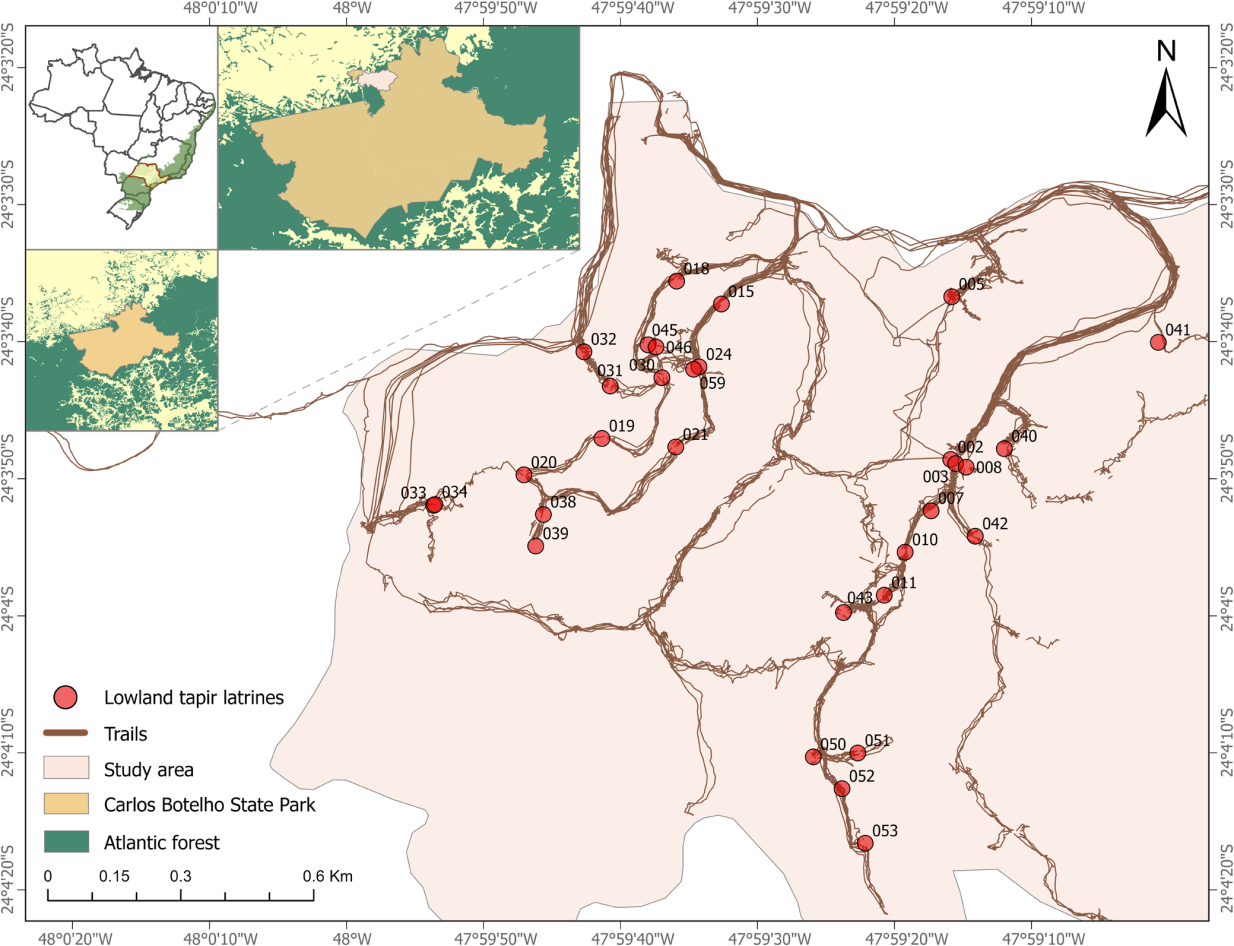
Distribution of lowland tapir latrines sampled within Carlos Botelho State Park, São Paulo, Brazil.

We conducted our study in Carlos Botelho State Park (hereafter CBSP; 37,664 ha; 24°08′ S, 47°58′ W), located in São Paulo state, southeastern Brazil. Situated in the Atlantic forest biome, this region has undergone significant anthropogenic impacts, including deforestation, fragmentation, and defaunation (Galetti et al. 2021). Currently, the Atlantic forest retains < 30% of its original cover, of which only 10% is protected (Vancine et al. 2024). The CBSP forest is part of the continuous “Serra de Paranapiacaba” massif (360,000 ha), one of the largest remaining patches of this rainforest biome. The site has an average annual temperature ranging between 15°C and 19°C, with a total annual precipitation of 1500–2000 mm year^−1^. Its rugged terrain features slopes of up to 50° and elevations ranging from 30 to 1100 m above sea level. The vegetation is classified as Ombrophilous Dense Atlantic Forest, transitioning from lowland to montane physiognomies (Oliveira‐Filho and Fontes 2000). This forest comprises mature primary and secondary successional stages, supporting a rich diversity of flora with over 1100 documented vascular plant species across 525 genera and 140 families. Trees from the Lauraceae, Myrtaceae, and Arecaceae families are particularly abundant (Lima et al. 2011). Our study was conducted in an 850‐ha area at the northernmost edge of CBSP, a site previously identified as having a high likelihood of containing lowland tapir latrines based on earlier surveys (Bueno et al. 2013; Lautenschlager et al. 2024; Figure 1).

### 2.2 Sample Collection and Plant DNA Processing

The lowland tapir latrines were located, classified, and their coordinates recorded during searches of the study area from December 2021 to June 2022, with a total monthly search effort of 17 km walked. We classified the latrines into three discrete categories according to feces decomposition stage: “new” = latrines with fresh feces with a well‐defined and compact fecal balls; “semi‐new” = latrines that were deteriorated due to the abiotic and biotic factors, still with discernible fecal balls; and “old” = deteriorated latrines in which fecal balls were not discernable (Figure S1). In each latrine, we measured the latrine's length, width, and maximum height (cm) to estimate the surface area (Table S2). Only latrines with fresh fecal material (“new” latrines) were sampled to ensure high‐quality DNA, as degradation in older samples reduces PCR amplification success and taxonomic resolution. In each of 31 latrines, we collected three small samples (approximately 2 cm^3^) from different parts of the fecal mount. Samples were collected with sterile tweezers and gloves to prevent any contamination and contact with the soil. The samples were placed in sterile 50 mL plastic tubes filled with absolute ethanol (CH_3_CH_2_OH, P.M. 46,07) and refrigerated until further manipulation.

We extracted plant DNA from fecal samples using the DNeasy Plant Mini Kit (Qiagen, Hilden, Germany) according to manufacturer instructions. Three mini‐barcode regions (*ITS‐Asteraceae*, *P6loop*, and *rbcL*) were used for plant species identification (Table S1). Complete protocols for DNA extraction, PCR amplification, sequence processing, and bioinformatics analyses (following Saranholi et al. 2024) are provided in the Supporting Information. After filtering and processing the data, we used online databases, such as the Brazilian and Atlantic Forest plant list (Flora e Funga do Brasil 2025), to curate and validate the Operational Taxonomic Units (OTUs) assignments. OTUs were assigned to the finest taxonomic level achievable based on sequence similarity and database matches, resulting in identification to species level for some OTUs, while others could only be resolved to genus or family level. We obtained a reference species list from the study site (CBSP) from Lima et al. (2011), with the plant taxonomic information recently updated, and from the online Catalog of Plants from Brazilian Protected Areas (Colli‐Silva et al. 2021). Synonyms were manually confirmed using the Plants of the World online database (POWO 2025). This updated CBSP plant list was used to match with the DNA metabarcoding remote blast table, resulting in possible OTUs that are specific to the study site. The Flora e Funga do Brasil database was downloaded and manipulated by using the R package “florabr” (Trindade 2024). All data manipulation and analyses after bioinformatics were performed using R software (R Core Team 2025).

### 2.3 Local Plant Diversity Sampling

To characterize flora diversity within CBSP for comparison with the species consumed by lowland tapirs, we randomly selected 15 latrines from the 31 previously sampled and established paired 3 m × 3 m plots at each location—one positioned near the latrine (~1 m, “nearby” plots) and another 20 m away (“control” plots). However, complete paired data were available for only 14 latrines, resulting in 28 plots (14 paired sampling locations) used in subsequent analyses. In each plot, we identified and quantified all plant species across various growth forms (e.g., palms, trees, shrubs, and herbs), targeting individuals 30–300 cm in height. Plants were identified to the highest taxonomic resolution possible (species, genus, family, or morphotypes).

### 2.4 Plant Functional Traits

With the list of plant species consumed by the lowland tapirs (through the DNA metabarcoding) and the species composition from the vegetation plots, we selected five plant functional traits (SLA, LDMC, leaf nitrogen content, leaf thickness, and width) that are related to herbivore dietary preferences and LES (Potter et al. 2022). Plant trait data were obtained from the TRY Plant Traits repository (Kattge et al. 2020). Given the uneven trait availability across plant species, we applied a representativeness filter, ensuring that every trait was available for at least 20% of the species across both datasets. This filtering yielded 29 and 28 plant species with sufficient trait coverage from the DNA and plot datasets, respectively.

### 2.5 Data Analyses

To verify that our sampling effort was sufficient to characterize the diet of lowland tapirs, we used species rarefaction curves (Gotelli and Colwell 2001). Rarefaction curves involve selecting a specified number of samples that is equal to or less than the number of samples in the smallest dataset and randomly discarding reads from larger samples until the number of remaining reads is equal to this threshold (Willis 2019). We created rarefaction and sampling completeness curves based on: (A) consumed plant species richness, according to DNA metabarcoding refined results (i.e., after data curation and matching with the CBSP plant reference list), and (B) the species richness of each of the 31 sampled latrines, according to Hill diversity orders (*q*‐values), which quantify biodiversity in ecological communities (Figure S3). According to Hill numbers, *q* = 0 represents species richness, *q* = 1 corresponds to Shannon diversity, which weights species according to their abundance, and *q* = 2 represents Simpson diversity, which emphasizes evenness in the community by considering both community richness and proportions while emphasizing common species (Chao et al. 2014). All curves included sampling extrapolation showing the expected richness if sampling were continued, with 95% confidence intervals.

To check whether the composition of defecated plant species differs among the different latrines sampled, we calculated the pairwise dissimilarities in beta diversity between latrines using the Jaccard dissimilarity index (*β‐jac*) (Baselga 2010; Baselga and Orme 2012; Socolar et al. 2016). This index partitions beta diversity into turnover (*β‐tur*) and nestedness (*β‐nes*), where *β‐jac = β‐tur + β‐nes*. Turnover (*β‐tur*) is the proportion of species that are not shared among communities, and a high *β‐tur* reflects significant species turnover or replacement among communities, meaning that the species composition varies considerably between sites. The *β‐nes* component quantifies the dissimilarity due to differences in species richness among sites, where communities with fewer species are subsets of the species found in more diverse communities (Baselga and Orme 2012; Legendre and De Cáceres 2013).

We visualized the compositional dissimilarity of plant genera consumed by tapirs and those found in the field vegetation data (vegetation plots paired with the matching latrines) using Non‐metric Multidimensional Scaling (NMDS) analysis. NMDS is a statistical ordination technique that visualizes the similarities and differences in communities by accurately representing the observed data using fewer dimensions (Oksanen et al. 2025). We used the *Jaccard* dissimilarity matrix with k = 2 dimensions to visualize patterns in plant genus composition among latrines and vegetation plots, while also checking the performance of the ordination using the stress value. The ordination performance is categorized as poor if the stress is greater than 0.2, fair if it is between 0.1 and 0.2, and good when the value is less than 0.1 (Clarke 1993). To assess whether there were significant compositional differences between the genera present in the tapir dietary data and those in the surrounding vegetation plots, we used Permutational Multivariate Analysis of Variance (PERMANOVA) with 999 permutations performed using the *Jaccard* distance matrix.

Moreover, we complemented this analysis with a Mantel test to assess the relationship between geographic distances and dissimilarity in genus composition, using data on the diet of lowland tapirs and the surrounding vegetation. This approach allows us to determine whether spatial autocorrelation influences compositional patterns and whether both sampling approaches capture similar spatial structures in plant community composition. We maintained consistency by using the *Jaccard* dissimilarity index for presence‐absence data (Legendre et al. 2015). For both analyses, we used plant genera rather than species, as genus‐level identification is more reliable in field conditions and many plant specimens from our plots could not be accurately identified to the species level.

To compare the LES traits of plant species detected through tapir dietary data versus those recorded in vegetation plots, we conducted a Principal Component Analysis (PCA) using functional traits with good representativeness (≥ 20% coverage). To maintain the biological distinction between consumed and available plant species, each species was treated separately according to its detection method. When both methods detected the same species, both records were retained as independent observations to preserve all evidence of consumption while maintaining the contrast between tapir diet composition and local vegetation availability. This approach avoided the loss of biological information that would occur from the arbitrary prioritization of one dataset over another. Missing trait values were imputed using mean values to maximize species inclusion in the analysis. The PCA was performed on standardized trait values to account for different measurement scales across traits.

Finally, to assess whether tapirs selectively consume plant species with distinct functional trait profiles, we tested for significant differences between the two groups (dietary and plot data) using a PERMANOVA based on Euclidean distances in the complete trait space (999 permutations). We also tested the assumption of homogeneous multivariate dispersions between groups using the *betadisper* function, which measures the distance of each sample to its group centroid in multivariate space to assess whether groups have similar levels of within‐group variability. The results were visualized using a biplot, which shows the first two principal components, with trait vectors indicating the direction and magnitude of each trait's contribution to the components. We added 95% confidence ellipses around each group to illustrate the functional trait space occupied by each data source. This approach allowed us to assess whether tapirs selectively consume plant species with distinct functional trait profiles compared to the available plant community, and to examine which functional traits contribute most strongly to any observed patterns between consumed and available species (Potter et al. 2022). All analyses were performed using the R environment (R Core Team 2025) using the package “iNEXT” (Hsieh et al. 2024) for the species rarefaction curve, “vegan” (Oksanen et al. 2025) for the diversity analysis, NMDS ordinations, PERMANOVA, *betadisper*, and Mantel test, “factoextra” for PCA visualization, and “tidyverse” for data wrangling and visualization (Wickham et al. 2019).

## 3 Results

### 3.1 Lowland Tapir Diet

According to the metabarcoding results, the lowland tapir latrine samples contained a total of 61 distinct plant species, from 69 genera and 46 families (Table S3). At the species level, we found that tapirs mostly consumed *Centella asiatica* , *Bertolonia acuminata*, *Solidago chilensis*, *Cissampelos pareira* , and *Myrcia multiflora*. The most prevalent genera were *Centella, Cyathea, Solanum, Miconia*, and *Cissampelos*, and the most prevalent families were Melastomataceae, Asteraceae, Myrtaceae, Cyathaceae, Apiaceae, Rubiaceae, and Solanaceae (Figure S2). Regarding plant growth forms, tapirs primarily consumed trees (37.5%), followed by herbaceous plants (25%), shrubs and subshrubs (17%), climbers (13%), and tree ferns (7.5%). Most of the consumed plants are zoochoric (55%), followed by anemochoric (28%), hydrochoric (9%), autochoric (6%), and other dispersal syndrome types (2%). Our sampling rarefaction curves showed that our sampling effort has not reached complete species saturation, that is, there are likely additional rare and less common species that would be discovered with more extensive sampling. When analyzing the species diversity, dominant species communities were better characterized than the full species assemblages. Different latrines exhibit varying levels of species diversity and community structure, as indicated by the Shannon and Simpson curves (Figure S3).

### 3.2 | Latrines Compositional Differentiation

**FIGURE 2:**
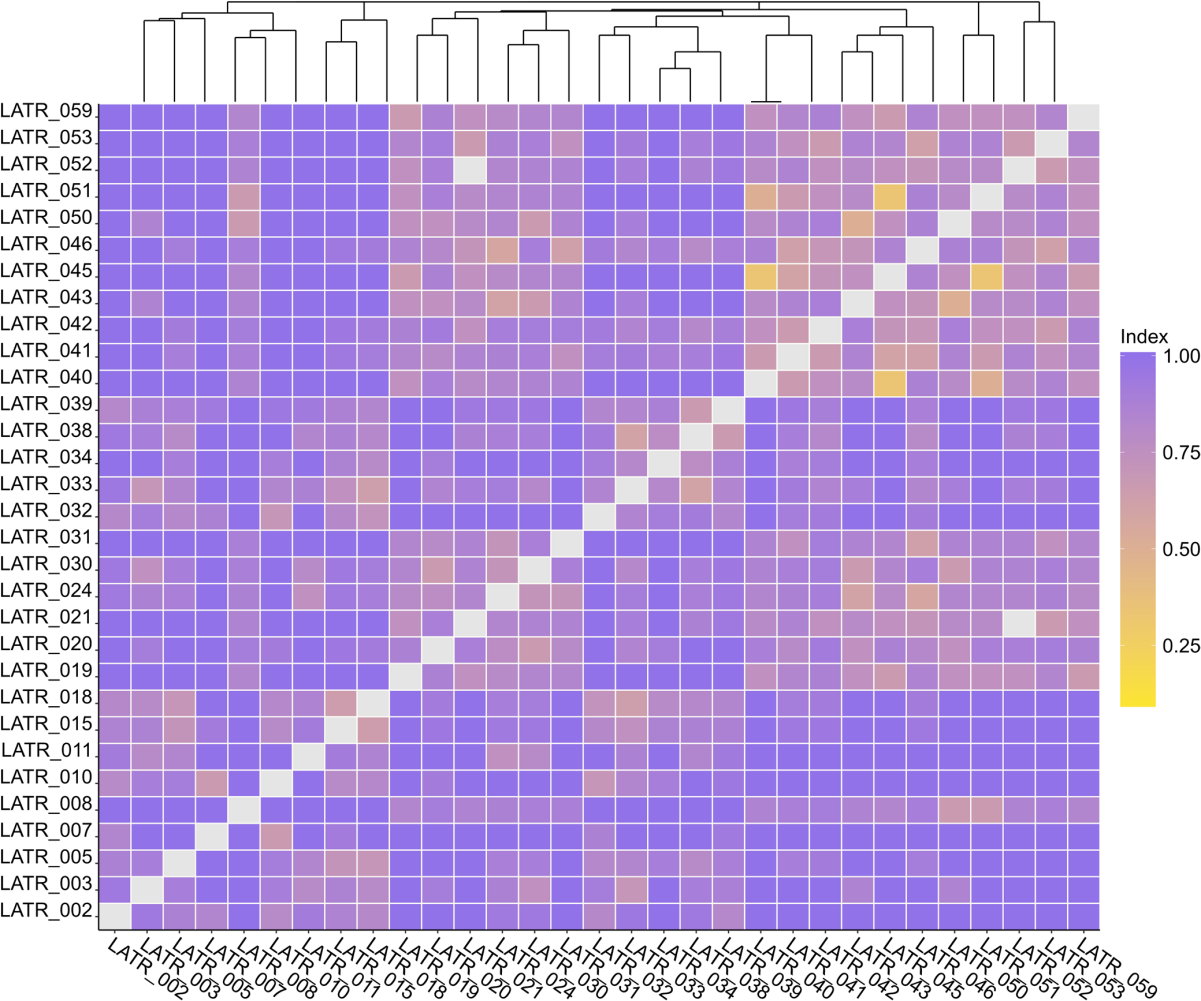
Results from the *Jaccard* beta diversity analyses for species richness, with pairwise dissimilarity matrices comparing tapir latrines (*β‐jac* diversity). High values of *β‐jac* index indicate that the latrines being compared had very different species composition (darker purple colors in the gradient indicate high values, ~1.00).

When we compared the plant composition of defecated plant species among the 31 latrines sampled, we found high beta‐diversity dissimilarity indexes (*β‐jac*, Figure 2). Overall, the spatial *β‐tur* component contributed the most to *β‐jac*, indicating that latrines are dissimilar due to a strong spatial turnover of species composition (*β‐tur*, Figure S4B) and low nestedness (*β‐nes*, Figure S4C). Most latrines exhibited a high dissimilarity index (~1.00), indicating that the samples being compared are distinct in terms of species composition.

### 3.3 Defecated Species Compared to Surrounding Vegetation

**FIGURE 3:**
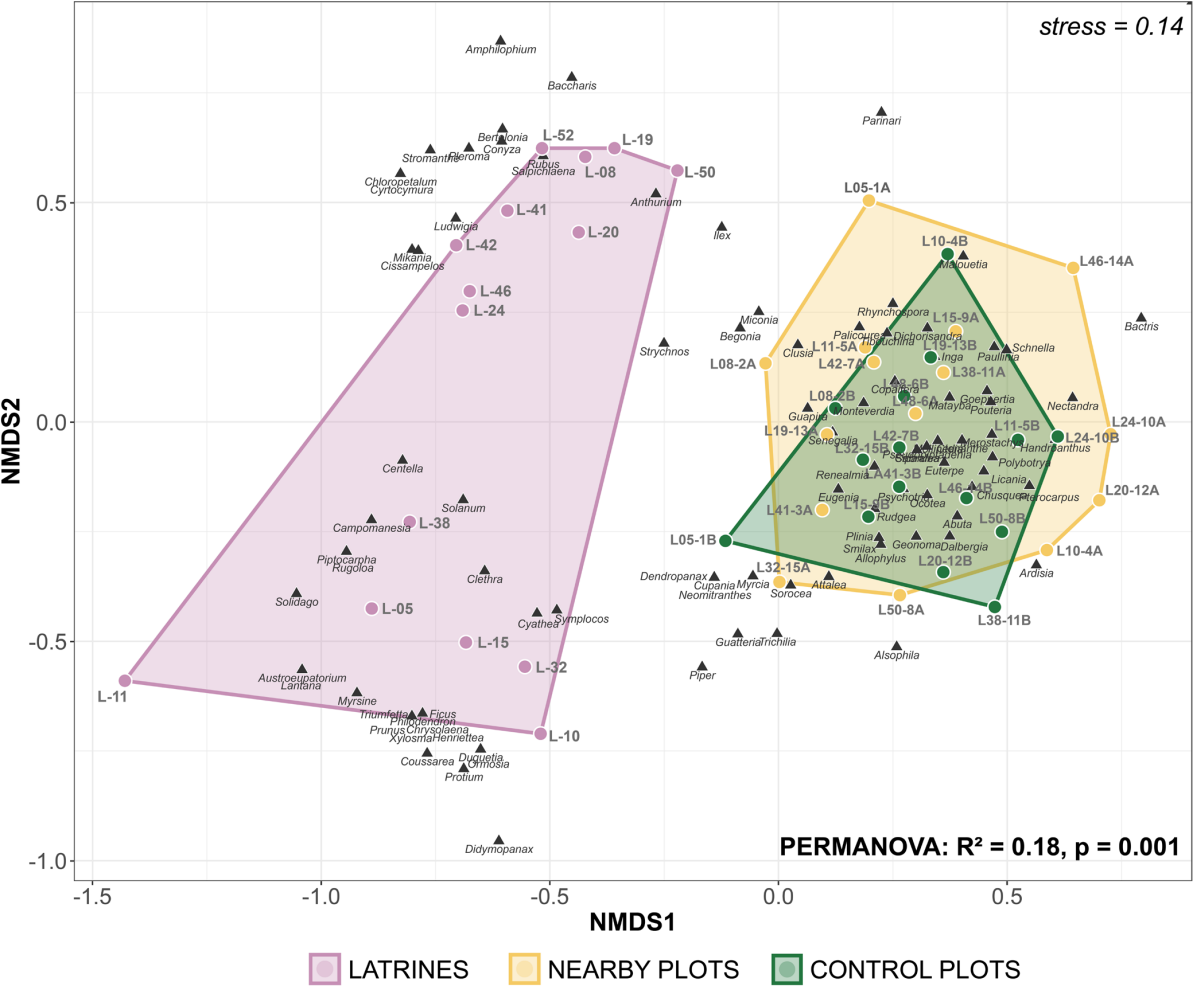
Dietary dissimilarity between the metabarcoding results and plot vegetation composition. NMDS ordination shows the distribution of plant genera based on community similarity. Each triangle represents a plant genus, with colors indicating different groups (dietary data from latrines in pink, nearby plots “A” in yellow (i.e., plots near latrines), and control plots “B” in green (i.e., plots away from latrines)). PERMANOVA revealed significant compositional differences among groups (*R* ^2^ = 0.18, *p* = 0.001), with the ordination showing acceptable fit (stress = 0.14).

Compositional analysis revealed significant differences between the dietary composition and the surrounding vegetation. Plant genera detected in tapir latrines differed substantially from those recorded in paired vegetation plots (PERMANOVA: *F* = 4.73, *R* ^2^ = 0.18, *p* = 0.001), indicating that tapirs do not consume plant genera according to their local availability. The overall goodness‐of‐fit for the entire ordination was considered fair (stress value > 0.1) (Figure 3). The Mantel test revealed no significant correlation in latrine and plot composition across space (*r* = 0.0186, *p* = 0.267). This suggests that the compositional differences between the tapir's diet and local vegetation are consistent across all geographic locations. The dissimilarity values (most points clustered between 0.6 and 1.0 on the *Jaccard* dissimilarity scale) indicate consistently high compositional turnover between diet and vegetation across all sampling sites (Figure S5).

### 3.4 Dietary Leaf Economic Spectrum Traits

Functional trait analysis revealed no significant differences in the LES traits between the plant species consumed by tapirs and those in the local vegetation. PCA captured 63% of the variation on the first two axes (39.7% on PC1 and 23.3% on PC2), indicating moderate structuring among the measured functional traits. PC1 was positively loaded on LDMC and leaf width, contrasting with SLA and nitrogen content (negatively loaded), representing the classic “slow‐fast” plant economics spectrum. PC2 captured additional morphological variation, with leaf thickness representing another axis of trait variation often linked to leaf toughness and shade tolerance. The SLA and nitrogen traits loaded together relate to fast‐end plant species, whereas LDMC and leaf width are traits associated with slow‐end plant species, which exhibit resource conservation, tougher leaves, and increased stress tolerance.

**FIGURE 4:**
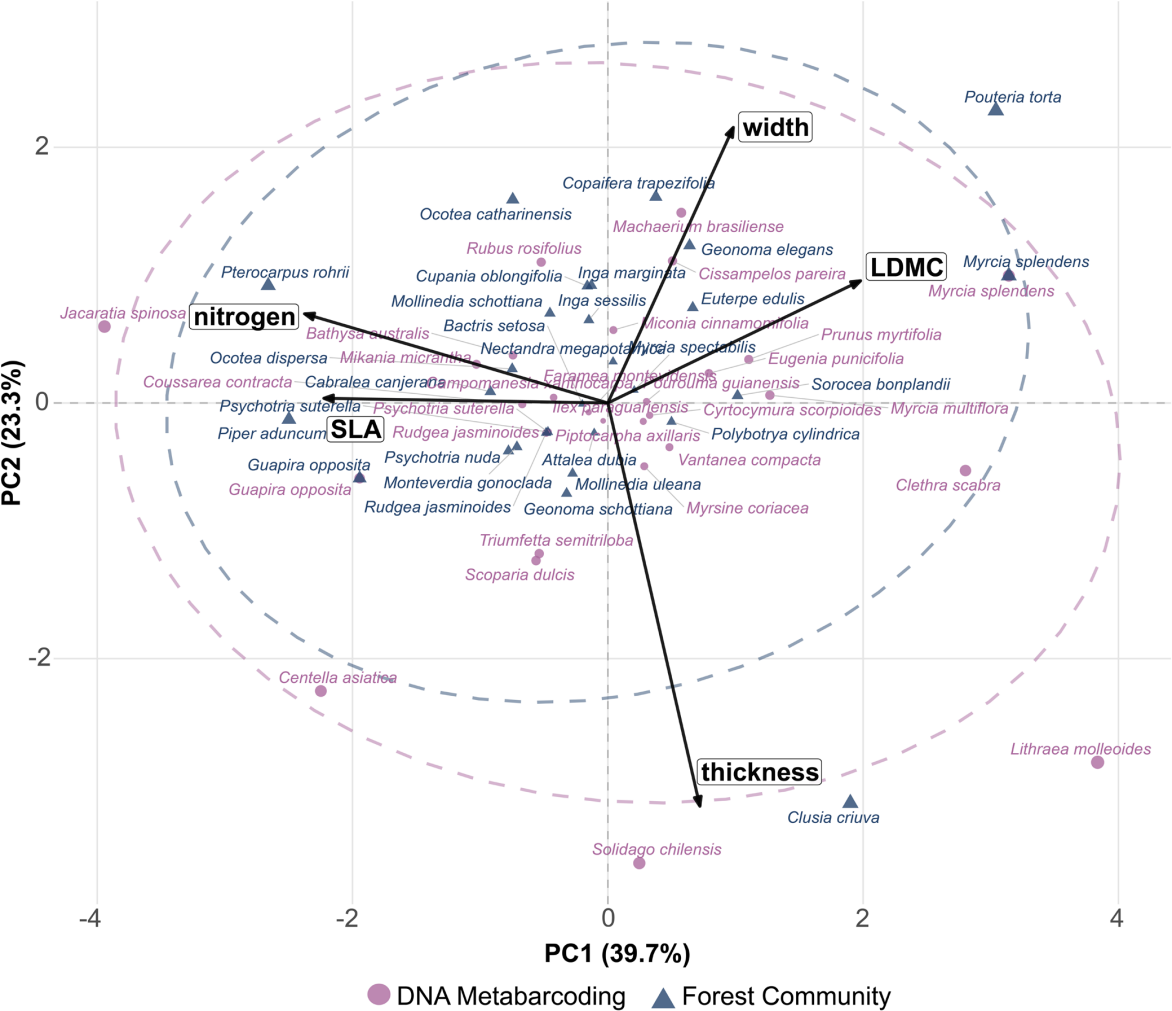
Position of plant species related to the lowland tapirs' diet and local vegetation composition along the LES traits. Plants consumed by tapirs in pink circles (DNA metabarcoding data) and plants from local vegetation in blue triangles (plot data). Black arrows show plant trait loadings, with arrow length indicating the strength of each trait's contribution to the principal components. Dashed ellipses represent 95% confidence intervals.

Our PCA shows substantial overlaps between the confidence ellipses for the consumed species and local vegetation, with both groups spanning similar regions of trait space. The PERMANOVA analysis confirmed that the tapirs' dietary composition did not differ significantly from that of the available vegetation in terms of LES traits (*F* = 1.08, *R* ^2^ = 0.019, *p* = 0.368). The *betadisper* test results were also non‐significant, revealing no significant differences in dispersions between groups (*F* ^1,55^ = 0.55, *p* = 0.488, 999 permutations), confirming that the assumption of homoscedasticity was met, thus validating the use of PERMANOVA for testing group differences in multivariate trait composition. Therefore, our results suggest that there is minimal selective feeding based on these LES traits. While some consumed species appear in regions associated with higher SLA and nitrogen content (fast‐end strategy), others cluster with species characterized by higher LDMC and leaf width (slow‐end strategy) (Figure 4).

## 4 Discussion

We used DNA metabarcoding to conduct a novel and detailed investigation into the diet of the largest terrestrial herbivore in South America, the lowland tapir. In an undisturbed Atlantic forest, tapirs consumed a diverse range of plant species from many genera and taxonomic families, particularly Melastomataceae, Asteraceae, and Myrtaceae—dominant, species‐rich families that play crucial roles in forest structure and dynamics (Souza et al. 2022). Most of the consumed species are zoochoric, relying on vertebrates for seed dispersal and recruitment (Howe and Smallwood 1982). Although we are unable to know which vegetative parts were consumed by tapirs using DNA metabarcoding, tapirs are known to consume fruits from approximately 300 species across 66 families, highlighting their importance as seed dispersers (Paolucci et al. 2019), also contributing to sustaining the local vertebrate community through their defecation sites (Lautenschlager et al. 2024). We recognize that in such species‐rich forests, identifying all plant individuals is often a difficult and subjective task and that barcoding sequences for the Atlantic forest tree flora are incomplete. However, Lima et al. (2018) demonstrated that approximately 90% of tree species in typical Atlantic forest surveys are represented in DNA barcoding reference libraries, enhancing the reliability of plant identification in ecological and dietary studies.

The high diversity of consumed plants also reflects the generalist habit of lowland tapirs. Composition in spatially separated latrines was highly dissimilar, resulting in a high turnover (*β‐tur*) of defecated plant composition among latrines, indicating distinct species composition between communities. High dissimilarity was also detected when comparing plant genera in latrines with surrounding vegetation, suggesting that lowland tapirs do not consume plants solely according to local availability. Instead, tapirs potentially forage across large areas with heterogeneous plant communities rather than simply consuming the most abundant local plants. With extensive home ranges averaging 8.31 km^2^ (Medici et al. 2022), lowland tapirs broaden their repertoire of consumed plants, later defecated in communal latrines. Lowland tapirs share these latrines with other individuals for many years, reflecting the varied dietary preferences within the population (Acosta et al. 1996; Fragoso 1997).

Our results support the hypothesis that lowland tapirs are generalist feeders consuming numerous plant species irrespective of plants' resource acquisition strategies. We found no indication that dietary preferences were related to leaf traits or position on the LES, despite some consumed species having high SLA and nitrogen content. High SLA relates to fast‐growing species with higher photosynthetic rates and less leaf structure investment (Wright et al. 2004; Donovan et al. 2011), correlating with herbivore preference due to softer texture and higher nutrient content (Zhu et al. 2024). Higher nitrogen concentrations generally enhance leaf palatability and nutritional quality, promoting herbivory (Mattson 1980). However, tapirs also consume plant species characterized by higher LDMC and leaf width (the slow end of the LES). Leaf thickness and width serve as important structural traits, indicating a greater investment in leaf material (Onoda et al. 2017). This thickness may provide mechanical advantages for the plant, offering protection against herbivory and physical damage, while wider leaves may enhance photosynthetic efficiency by maximizing light capture (Hanley et al. 2007; Lusk et al. 2019). Plant species with a high LDMC not only imply denser leaf material but could also correlate with higher concentrations of secondary metabolites, which usually discourage herbivores from foraging on them (Carmona et al. 2011; Blumenthal et al. 2020). Tapirs, however, seem to disregard this, as they consume plant species with such traits. The spread of traits along both PC axes (explaining 63% of variation) indicates that tapirs handle diverse plant textures and nutritional profiles, browsing from tough to softer, more digestible leaves. Their hindgut fermentation system enables the efficient processing of low‐quality foods (Hibert et al. 2011), allowing them to consume a wide variety of plant species with diverse functional traits.

The lack of clear functional trait‐based selectivity may indicate that other factors, not captured by the traits we selected, such as secondary compounds or nutritional content beyond nitrogen, drive tapir foraging decisions. It is also important to highlight the challenges associated with using large‐scale data from global ecological databases, such as the TRY database (Augustine et al. 2024). The intraspecific variation in traits across the same species at different locations, as well as the low availability of tropical plant species data for the five functional traits we selected, might underestimate the true extent of lowland tapirs' dietary selection and preferences. For our analysis, we used only half of the known consumed plants and species from the vegetation community, based on data availability and the representativeness of each species for its respective trait. Moreover, since we cannot determine which stage of the plant is being consumed (e.g., fruits, seeds, seedlings, saplings, or adults) using the metabarcoding approach, we assumed that all species were adults to obtain their LES traits and provide a first glimpse into plant consumption and trait selection by lowland tapirs in tropical forests. Still, we acknowledge that each developmental stage can have distinct morphological, anatomical, and biochemical traits (Vitória et al. 2019). Future studies should therefore incorporate plant developmental stages and ontogenetic variation when assessing herbivore dietary preferences using functional traits, to fully understand foraging strategies. Additionally, integrating both vegetative (leaves) and reproductive (fruits) plant traits would provide a more complete understanding of tapir dietary ecology, especially considering the seasonal importance of fruits and their role in seed dispersal networks.

Large herbivorous mammals range along a continuum from grazers that primarily consume monocots (e.g., grasses) to browsers that feed on woody foliage, with mixed feeders flexibly consuming both, allowing them to thrive under varying conditions (Venter et al. 2019). Ungulate diets are shaped by habitat and forage availability. Many studies have focused on African ungulates, their niche partitioning, and the impacts on their ecosystem (Pansu et al. 2019; Kartzinel and Pringle 2020; Potter et al. 2022). In African savannas, morphological traits (e.g., body size, mouth width, dentition, digestive system) align with plant traits (e.g., height, leaf size, structural and chemical components), driving differential plant use and spatial foraging, and promoting coexistence (Pansu et al. 2022). Tropical mammalian herbivores are generally smaller‐bodied. The remaining community, including deer species (Cervidae), peccaries, and lowland tapirs, exhibits diverse dietary strategies shaped by high plant diversity and habitat complexity. The grazer‐to‐browser continuum also occurs in hyperdiverse forests, where some species specialize on rarely consumed plant families (Mata et al. 2024). Recent metabarcoding studies further reveal spatial dietary variation among large Neotropical herbivores, emphasizing their role in ecosystem functioning (Barreto et al. 2025). Still, their low abundance in tropical forests makes dietary studies and niche overlap assessment challenging.

## 5 Conclusion

Our findings support the hypothesis that the lowland tapir's diet can promote a high turnover of plants by consuming species that are not only found locally, thereby helping to maintain plant diversity. Although we cannot determine which plant parts were consumed using only the DNA metabarcoding approach, more than 50% of the species exhibit zoochoric dispersal syndromes, highlighting the seed dispersal role of lowland tapirs, as they are the last potential dispersers of Neotropical plant species with large seeds that were formerly dispersed by extinct megafauna. Lowland tapirs' generalist habit contributes to maintaining local plant diversity, as they consume species spanning both the fast and slow ends of the LES. The high diversity and abundance of tropical forest plants favor generalist herbivores, as they do not necessarily exhibit a preference for specific functional traits, groups, or species. Comparable studies are needed across different ecosystems to improve our knowledge of large herbivores' dietary selection, particularly for threatened megafauna. Lowland tapir populations face numerous anthropogenic threats, including habitat loss, fragmentation, poaching, road collisions, pollution, disease, and harassment from domestic dogs (Medici and Desbiez 2012). An integrated conservation framework is essential to protect both tapir populations and their wide‐ranging habitat needs, as the loss of this important large herbivore could result in ecological cascades that decrease plant diversity and composition in tropical forest communities.

## Author Contributions

**Laís Lautenschlager:** conceptualization (lead), data curation (lead), formal analysis (lead), funding acquisition (lead), investigation (lead), methodology (lead), project administration (lead), resources (lead), software (lead), supervision (equal), validation (lead), visualization (lead), writing – original draft (lead), writing – review and editing (lead). **Yuri Souza:** conceptualization (supporting), data curation (supporting), formal analysis (equal), methodology (supporting), software (supporting), visualization (supporting), writing – original draft (supporting), writing – review and editing (supporting). **Luísa Genes:** conceptualization (supporting), writing – original draft (supporting), writing – review and editing (supporting). **Bruno H. Saranholi:** data curation (supporting), investigation (supporting), methodology (supporting), software (supporting), validation (supporting), writing – original draft (supporting), writing – review and editing (supporting). **Carla C. Cristina Gestich:** data curation (supporting), methodology (supporting), software (supporting), validation (supporting), writing – original draft (supporting), writing – review and editing (supporting). **Carina I. Motta:** data curation (supporting), methodology (supporting), writing – original draft (supporting), writing – review and editing (supporting). **Valesca B. Zipparro:** methodology (supporting), writing – original draft (supporting), writing – review and editing (supporting). **Pedro Galetti Jr.:** investigation (supporting), methodology (supporting), project administration (supporting), resources (supporting), writing – original draft (supporting), writing – review and editing (supporting). **Mauro Galetti:** funding acquisition (equal), investigation (supporting), methodology (supporting), project administration (supporting), resources (supporting), supervision (supporting), writing – original draft (supporting), writing – review and editing (supporting). **Kenneth J. Feeley:** funding acquisition (supporting), investigation (supporting), project administration (supporting), resources (supporting), supervision (equal), writing – original draft (supporting), writing – review and editing (supporting).

## Disclosure

Our study brings together authors from various countries, including scientists based in the country where the study was conducted. All authors were engaged early on with the research and study design to ensure that the diverse sets of perspectives they represent were considered from the onset. Whenever relevant, literature published by scientists from the region was cited; efforts were made to consider relevant work published in the local language.

## Ethics Statement

All research activities were conducted under permits issued by the “Biodiversity Authorization and Information System” (SISBIO, #80284‐1 in 2022, and #89209‐1 in 2024), managed by the “Chico Mendes Institute for Biodiversity and Conservation” (ICMBio), within the Brazilian Ministry of the Environment. SISBIO is the official federal platform that regulates and authorizes scientific research involving wild fauna, flora, and natural ecosystems in Brazil, including the collection, handling, and monitoring of biological material. Environmental DNA and genetic activities were registered in the “National System for the Management of Genetic Heritage and Associated Traditional Knowledge” (SisGen, A0BA7FA). Fieldwork in the study site was registered and authorized by the “Research Registration and Management System” (CadGP, #000000006578/2023, from 2023 to 2025) of the Secretariat of Environment, Infrastructure, and Logistics (SIGAM/SEMIL), the state‐level authority responsible for regulating scientific research and environmental management in São Paulo, Brazil.

## Conflicts of Interest

The authors declare no conflicts of interest.

## Supporting information


**Data S1:** Supporting information.

## Data Availability

The main datasets supporting the analyses are available at “20260130_SuppInfo_Lautenschlager_etal_Ecology&Evolution_v1” on FigShare https://doi.org/10.6084/m9.figshare.30214249.
