# Supplementary material for: Unveiling the Dietary Selection of Lowland Tapirs (Tapirus terrestris) in a Tropical Rainforest
Source: Ecol Evol. 2026 Feb 25;16(3):e73161. doi: 10.1002/ece3.73161 (PMC12935466; doi:10.1002/ece3.73161)
Supplement: Supplementary file 1 — Data S1: Supporting information. [file ECE3-16-e73161-s001.docx]

Title: ***Unveiling the dietary selection of Lowland tapirs (Tapirus terrestris) in a tropical rainforest***

***Supporting Information***

***PCR Amplification and DNA Metabarcoding sequencing processing***

DNA extractions were performed at the Laboratory of Molecular Ecology, Department of Biodiversity, São Paulo State University (Universidade Estadual Paulista – UNESP) in Rio Claro, São Paulo, Brazil. Because fecal samples contained up to 50 ml of material, three subsamples were taken from each fecal sample to improve the detection of diet items (Saranholi, *pers. comm*., 2024). The three small subsamples (20 to 50 mg, fresh mass) were separated from the top (A), middle (B), and lower (C) portions of each fecal sample using sterilized tweezers and placed in 2 mL autoclaved polypropylene microtubes for extraction. To each subsample, 200 µL of the alcohol used to store the fecal sample was added, yielding 20–50 mg of fecal material plus 200 µL of alcohol per subsample. Samples were dried under a fume hood in a dry bath (65 °C) for two hours or until the alcohol evaporated. Following drying, DNA was extracted using a DNeasy Plant Mini Kit (Qiagen, Germany) following the Quick-Start Protocol, with the omission of RNase treatment. DNA extractions were performed using appropriate personal equipment (gloves and mask), and surfaces were sterilized with a 10% bleach solution.

We used DNA metabarcoding to identify the plant diet contents of the lowland tapir fecal samples, utilizing the *ITS-Asteraceae*, *P6loop*, and *rbcL* mini-barcodes (*Table* ***S1***), which are widely recognized for amplifying plant barcodes. To obtain greater species-level identification, we employed a combination of markers. The suitability of the *P6loop* region for fecal metabarcoding is due to its high conservativeness of primer sequences, short fragment size, and moderate variation. This barcode marker has been demonstrated to perform more effectively in terms of plant detection and quantification through fecal metabarcoding (Mallott et al., 2018). We also used taxon-specific markers, such as ITS (*ITS-Asteraceae*), and *rbcL*, a widely used molecular marker sourced from chloroplast DNA, which is particularly instrumental in plant identification and phylogenetic studies (Newmaster et al., 2006; Erikson et al., 2017).

Polymerase Chain Reactions (PCRs) were conducted within a UV-sterilized hood in a dedicated PCR room. Unique identifiers (tags) obtained from Axtner et al. (2019) - TagA: TGCAT, TagB: TCAGC, TagC: AAGCG - were added to both forward and reverse primers to label each PCR amplicon, allowing us to individualize the three subsamples of each fecal sample (e.g., Saranholi et al., 2024). The PCR protocol followed: 1x buffer (Tris–HCl 20 mM, pH 8.4, and KCl 50 mM), 0.5 mM of each primer, 0.2 mM dNTP (Invitrogen), 2.5 mM MgCl_2_, 0.8mg/mL BSA (Bovine Serum Albumin), 1.0 U, Platinum Taq polymerase (Invitrogen), and 3 μL of template DNA. Cycling conditions were 10 min at 95 °C, followed by 40 cycles of 30 s at 95 °C, 30 s at 50 °C (*P6loop* and *ITS1Ast*) or 52°C (*rbcL*), and 30 s at 72 °C, with a final extension of 10 min at 72 °C. To check for contamination, PCR amplifications included a non-template sample as a negative control. The amplified products were visualized on 1.5% agarose gels by electrophoresis. A second PCR was performed for samples that failed in the first one, as a new amplification attempt. Each sample pool was built into a sequencing library. Libraries were built using the TruSeq DNA PCR-Free Library Preparation Kit (Illumina). Sequencing (paired-end) was performed at EcoMol Consultancy Lab Company in São Paulo, Brazil (see [https://ecomolconsultoria.com.br](https://ecomolconsultoria.com.br/)).

Bioinformatics steps and first dataset cleaning were performed with the assistance of Dr. Bruno Saranholi and Dr. Carla Gestich. The resulting sequences were demultiplexed using process_radtags in Stacks v2.59 (Catchen et al., 2013), where the identifier barcodes (tags) were used to trace the information back to each fecal sample replicate (Axtner et al., 2019). At this step, the barcode option inline–inline was used to eliminate misassignments caused by occasional tag-jumping events, which could result in incorrect matching forward and reverse tag sequences (Axtner et al., 2019). For the reads obtained from each fecal sample replicate, we used PEAR v.0.9.11 (Zhang et al., 2014) to merge the correspondent forward and reverse sequences and trimmed them to a minimum quality score threshold (−q) of 15, a minimum overlap (−v) of 50 base pairs and a minimum length (−n) of 50 base pairs. Primers were removed by suing cutadapt (Martin, 2011). Finally, we clustered the operational taxonomic units (OTUs) for the reads of each mini barcode separately for each tagged sample using USEARCH (Edgar, 2010), considering a 97% similarity among sequences and discarding all singletons from the analysis. The final OTU sequences were identified against GenBank (<https://www.ncbi.nlm.nih.gov/genbank/>) for plant species identification.

Several steps were taken to minimize uncertainty in our DNA matching. Initially, random records (e.g., fungi records, unclassified species, names with special characters or numbers) were removed from the data. The query coverage per subject and the percentage of identical matches were defined as ≥ 70% and ≥ 98%, respectively, for inclusion in the analysis. To limit sample contamination, we excluded any sequence detections that had fewer than 20 reads in a sample, or which constituted less than 0.05% of the total sequence reads per sample. This cutoff is recommended to balance the removal of false positives, and the retention of target reads (e.g., Saranholi et al., 2023, 2024; Barreto et al., 2025). All sequences found in the negative controls were removed from samples within the same sequencing pool.

***Supporting Information - Figures***

**
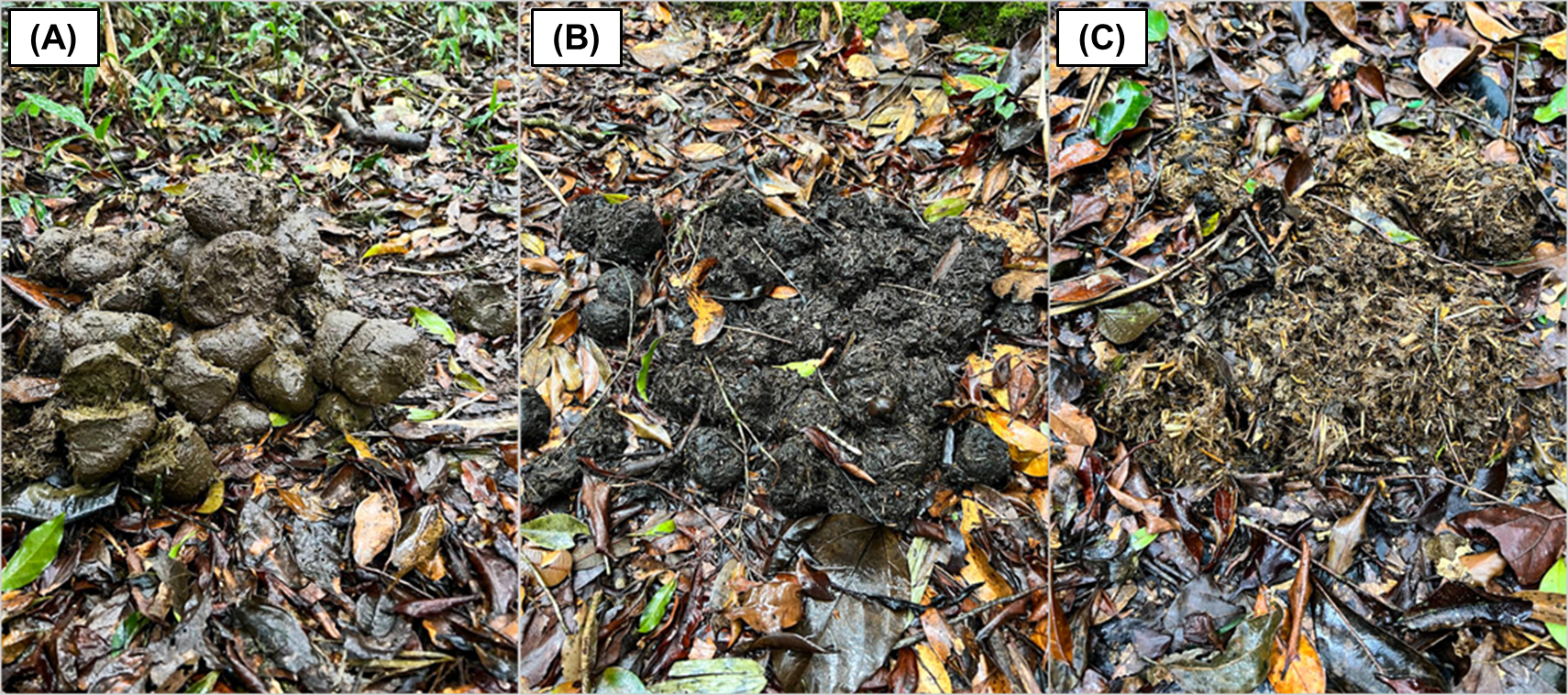
**

Figure **S1**. Lowland tapir (*Tapirus terrestris*) latrines in different stages of decomposition. According to our classification, (A) represents a "new" (fresh) latrine; (B) a "semi-new"; and (C) an "old" (deteriorated) latrine. All samples for DNA metabarcoding analyses were collected from new latrines.


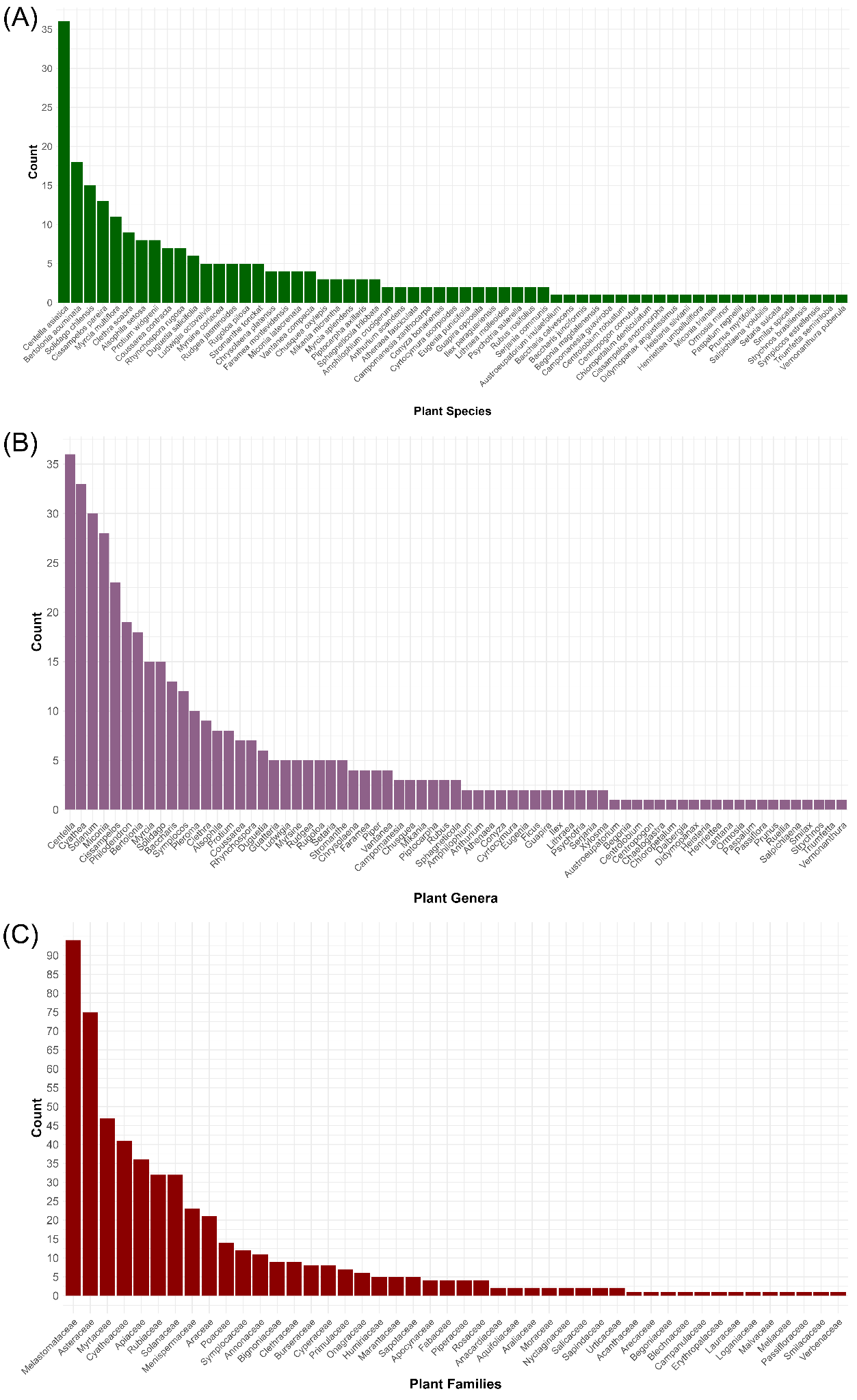


*Figure* ***S2***. Consumed plant species (A), genera (B), and families (C) according to DNA metabarcoding after quality filtering. Counts represent the number of OTUs in which these plants were identified within all 31 samples (lowland tapir latrines) for each taxonomic level.

**
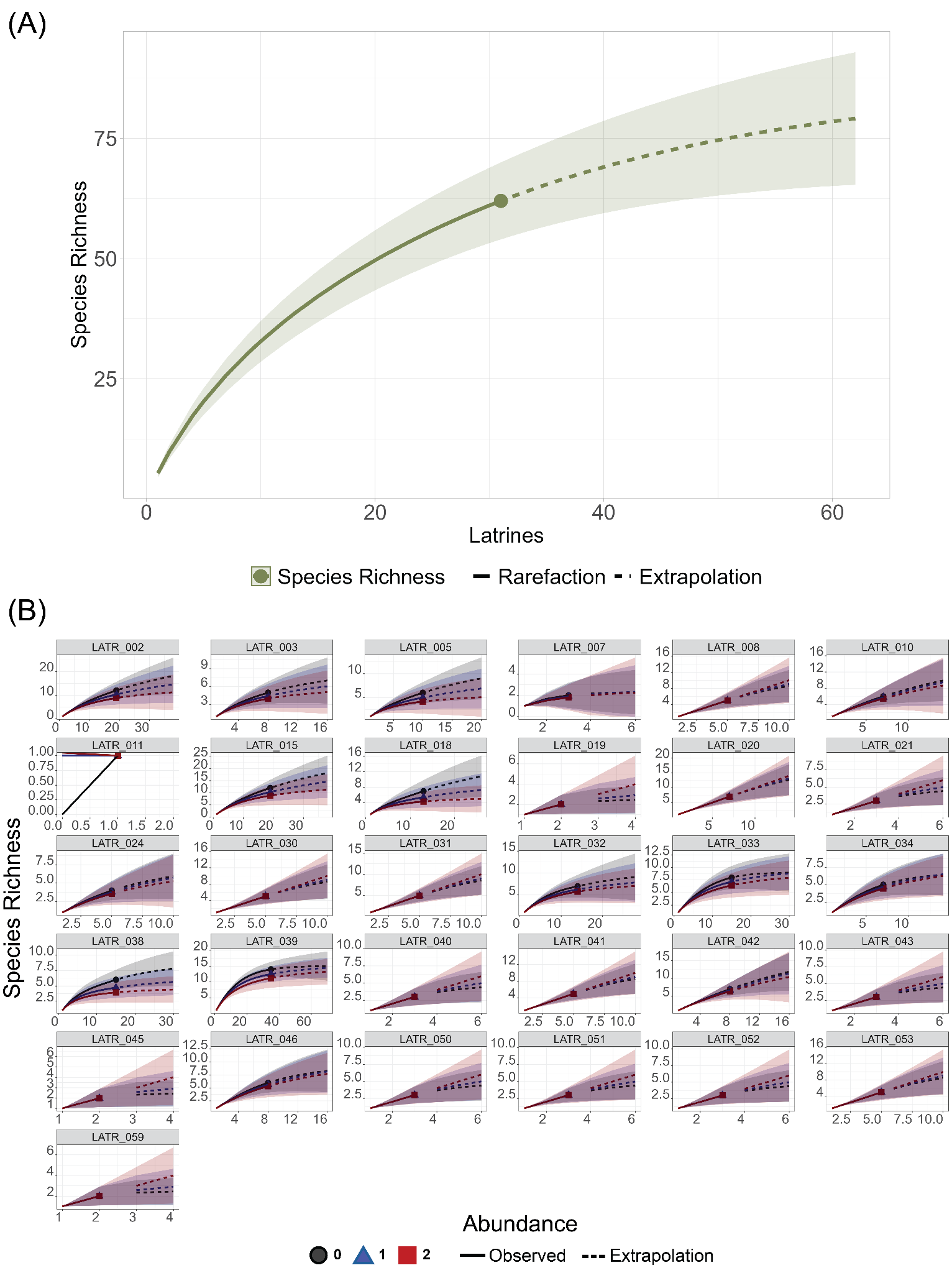
**

*Figure* ***S3***. Rarefaction and sampling completeness curves based on consumed plant species richness, according to DNA metabarcoding refined results (i.e., after data curation and matching with the study site's plant reference list). The curves include sampling extrapolation with the expected richness if sampling were continued. The completeness curve also shows the extrapolated effort required to achieve higher confidence intervals (> 95%). (A) Consumed species richness from all samples (latrines) combined. (B) Species richness of each of the 31 sampled latrines, according to Hill diversity orders (q-values): q = 0 (black) represents species richness (counts all species equally, regardless of abundance), q = 1 (dark blue) represents Shannon diversity (emphasizes common species while still considering rare ones), and q = 2 (dark red) represents Simpson diversity (weights dominant species, less sensitive to rare species).


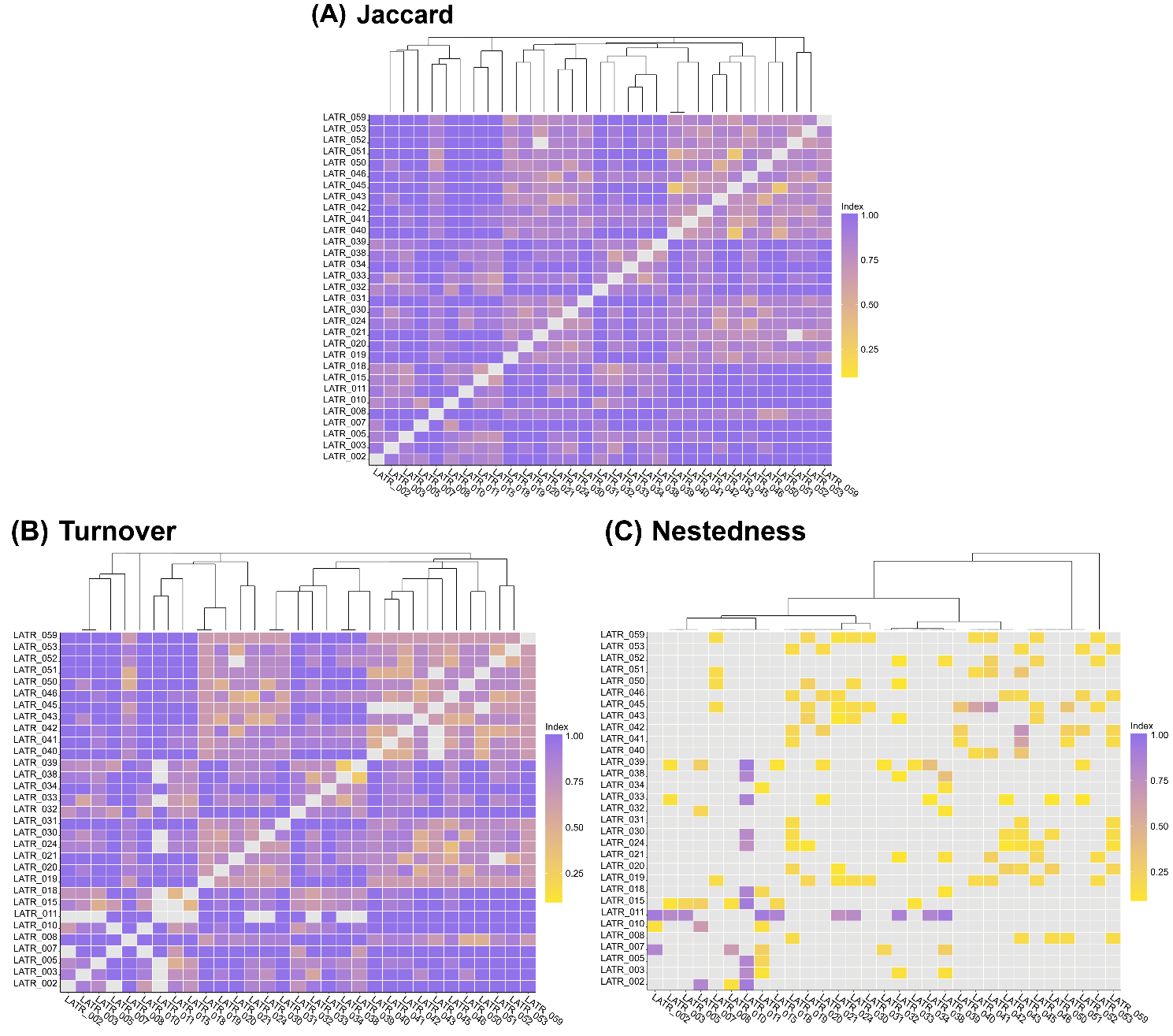


*Figure* ***S4***. Results from the Jaccard beta diversity analysis for species richness, with pairwise dissimilarity matrices comparing tapir latrines. *β-jac* (A), *β-tur* (B), and *β-nes* (C). The dendrogram at the top of each graph groups latrines based on their similarities on the x-axis of each graph. High values of *β-jac* indicate that the latrines being compared had very different species composition. High values of *β-tur* indicate significant turnover or replacement of species between communities. High values of *β-ness* indicate that communities with fewer species are subsets of the species found in more diverse communities. Darker purple colors in the gradient indicate high values (~ 1.00).


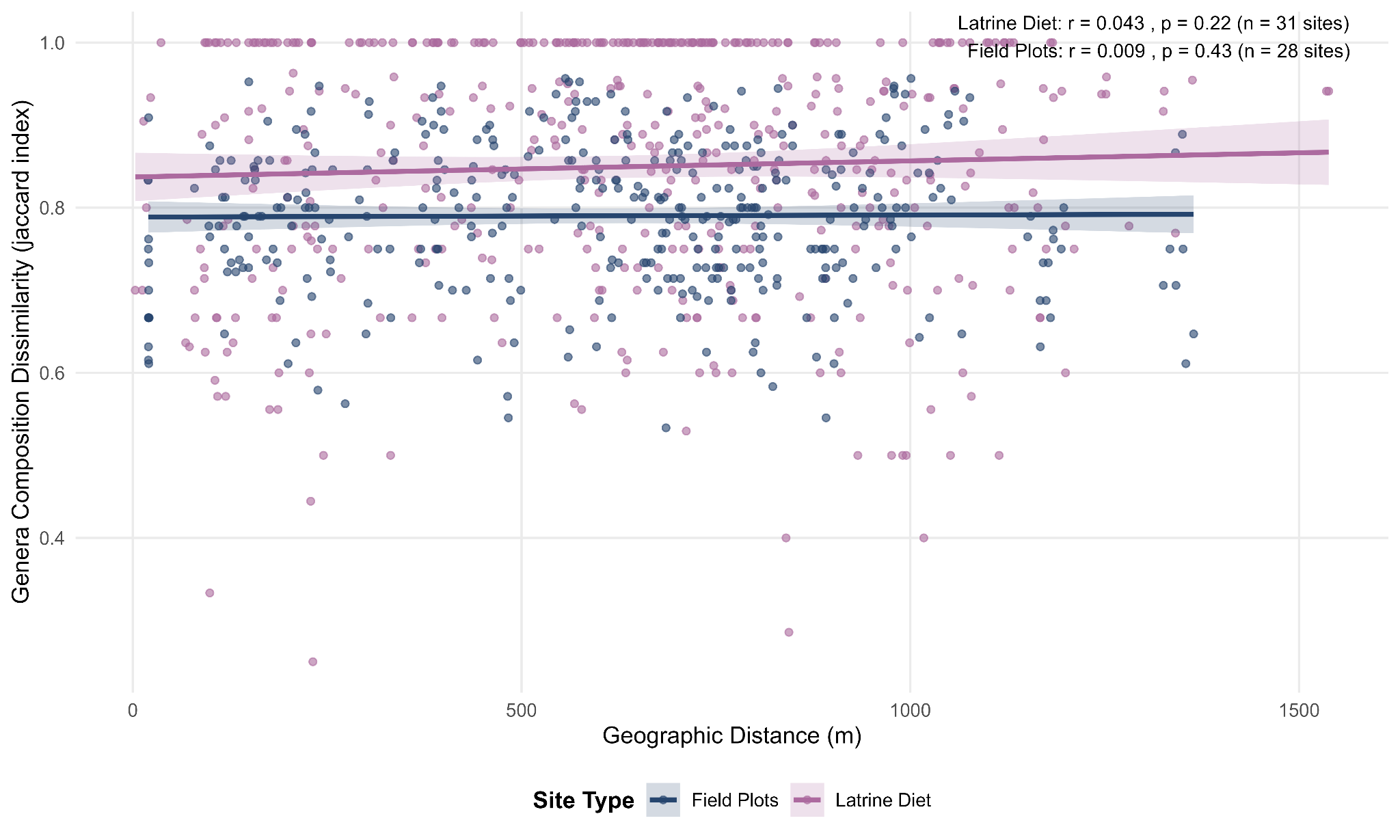


*Figure* ***S5***. Mantel test examining the relationship between geographic distance and dissimilarity in genus composition for the lowland tapir diet and local vegetation. Each point represents a pairwise comparison between sampling sites, with geographic distance (in meters) on the x-axis and the *Jaccard* dissimilarity index on the y-axis. Pink points represent dietary data (DNA metabarcoding, n = 31 latrines), blue points represent field plot vegetation data (n = 28 sites). Shaded areas indicate 95% confidence intervals. Neither dataset shows significant spatial autocorrelation (dietary data: r = 0.043, p = 0.22; field plots: r = 0.009, p = 0.43), indicating that genus composition dissimilarity is independent of geographic distance within the study area.

***Supporting Information - Tables***

*Table* ***S1***: Primers used for the mini-barcodes regions (*ITS-Asteraceae*, *P6loop*, and *rbcL*).

| **Primer Name** | **Sequence 5′–3′** | **Reference** |
| --- | --- | --- |
| P6loop_g | GGGCAATCCTGAGCCAA | Taberlet et al., 2007 |
| P6loop_h | CCATTGAGTCTCTGCACCTATC | Taberlet et al., 2007 |
| ITS1-F | GATATCCGTTGCCGAGAGTC | Ait Baamrane et al., 2012 |
| ITS1Ast-R | CGGCACGGCATGTGCCAAGG | Ait Baamrane et al., 2012 |
| rbcL1 | TTGGCAGCATTYCGAGTAACTCC | Palmieri et al., 2009 |
| rbcLB | AACCYTCTTCAAAAAGGTC | Palmieri et al., 2009 |

*Table* ***S2***. Summary information of 31 lowland tapir (*T. terrestris*) latrines sampled for obtaining DNA metabarcoding in Carlos Botelho State Park. Dates follow the US-centric MM/DD/YYYY structure.

| **Latrine**  **ID** | **Latitude** | **Longitude** | **Latrine Length (m)** | **Latrine Width (m)** | **Latrine Area (m^2^)** | **Date of collection** |
| --- | --- | --- | --- | --- | --- | --- |
| LATR_002 | -24.0635 | -47.987749 | 1.20 | 2.00 | 1.88 | 12/16/2021 |
| LATR_003 | -24.063583 | -47.987646 | 1.00 | 0.80 | 0.63 | 12/16/2021 |
| LATR_005 | -24.0602 | -47.987728 | 1.20 | 1.30 | 1.23 | 12/17/2021 |
| LATR_007 | -24.06454 | -47.988147 | 1.50 | 0.90 | 1.06 | 12/18/2021 |
| LATR_008 | -24.063655 | -47.987435 | 1.70 | 0.60 | 0.80 | 12/18/2021 |
| LATR_010 | -24.065378 | -47.988672 | 4.80 | 2.50 | 9.42 | 12/19/2021 |
| LATR_011 | -24.066251 | -47.989096 | 0.60 | 0.65 | 0.31 | 12/19/2021 |
| LATR_015 | -24.060348 | -47.992398 | 2.10 | 0.90 | 1.48 | 12/20/2021 |
| LATR_018 | -24.059885 | -47.993306 | 1.10 | 0.95 | 0.82 | 12/20/2021 |
| LATR_019 | -24.063072 | -47.994821 | 4.40 | 1.00 | 3.46 | 12/20/2021 |
| LATR_020 | -24.063811 | -47.996401 | 1.90 | 1.10 | 1.64 | 12/20/2021 |
| LATR_021 | -24.063244 | -47.993322 | 2.50 | 1.90 | 3.73 | 12/20/2021 |
| LATR_024 | -24.061622 | -47.992852 | 3.00 | 1.30 | 3.06 | 12/21/2021 |
| LATR_030 | -24.061839 | -47.993604 | 1.80 | 1.10 | 1.55 | 01/15/2022 |
| LATR_031 | -24.06201 | -47.994652 | 1.80 | 0.90 | 1.27 | 01/16/2022 |
| LATR_032 | -24.061314 | -47.995187 | 5.00 | 4.00 | 15.70 | 01/16/2022 |
| LATR_033 | -24.06443 | -47.998233 | 5.80 | 4.10 | 18.67 | 01/16/2022 |
| LATR_034 | -24.064412 | -47.998209 | 1.75 | 0.70 | 0.96 | 01/16/2022 |
| LATR_038 | -24.064614 | -47.996008 | 6.30 | 7.15 | 35.38 | 02/19/2022 |
| LATR_039 | -24.065253 | -47.996162 | 4.20 | 1.60 | 5.28 | 02/19/2022 |
| LATR_040 | -24.063279 | -47.986662 | 3.80 | 3.00 | 8.95 | 02/19/2022 |
| LATR_041 | -24.061126 | -47.98354 | 1.96 | 1.45 | 2.23 | 03/18/2022 |
| LATR_042 | -24.065054 | -47.987249 | 0.56 | 0.60 | 0.26 | 03/19/2022 |
| LATR_043 | -24.0666 | -47.989924 | 0.60 | 0.50 | 0.23 | 03/19/2022 |
| LATR_045 | -24.061174 | -47.993886 | 3.20 | 2.80 | 7.04 | 04/15/2022 |
| LATR_046 | -24.061221 | -47.993722 | 2.30 | 1.40 | 2.53 | 04/15/2022 |
| LATR_050 | -24.069526 | -47.990535 | 3.40 | 1.80 | 4.81 | 04/16/2022 |
| LATR_051 | -24.069447 | -47.989633 | 4.00 | 2.70 | 8.48 | 04/16/2022 |
| LATR_052 | -24.070172 | -47.989952 | 1.50 | 0.60 | 0.71 | 04/16/2022 |
| LATR_053 | -24.071278 | -47.989481 | 3.20 | 1.10 | 2.76 | 04/16/2022 |
| LATR_059 | -24.061669 | -47.992961 | 2.30 | 1.50 | 2.71 | 05/21/2022 |

*Table* ***S3***. List of families, genera, and species consumed by *T. terrestris* in Carlos Botelho State Park through analyzing the fecal DNA metabarcoding approach. Additional information includes the number of OTUs in which these plants were identified (counts) within all samples; the plant dispersal syndrome (Anemo = Anemochory, Auto = Autochory, Epizoo = Epizoochory, Hydro = Hydrochory, Zoo = Zoochory); plant growth form (B = Bamboo, C = Climber, G = Grass, H = Herb, S = Shrub, SS = Subshrub, T = Tree, TF = Tree Fern), and shade tolerance (Yes- tolerant, No- intolerant), according to the ReFlora database (see<https://reflora.jbrj.gov.br/>), the Global Biodiversity Information Facility (GBIF, see <https://www.gbif.org/>), or Plants of the World Online (POWO, <https://powo.science.kew.org/>) databases.

| **Family** | **Genus** | **Species** | **Counts  (OTUs)** | **Dispersal  syndrome** | **Growth  form** | **Shade  tolerance** |
| --- | --- | --- | --- | --- | --- | --- |
| Acanthaceae | Ruellia | *Ruellia* sp. | 1 | Auto | H | No |
| Anacardiaceae | Lithraea | *L. molleoides* | 2 | Zoo | S, T | No |
| Annonaceae | Duguetia | *D. salicifolia* | 6 | Zoo | T | Yes |
| Annonaceae | Guatteria | *Guatteria* sp. | 5 | Zoo | S, T | Yes |
| Apiaceae | Centella | *C. asiatica* | 36 | Hydro | H | Yes |
| Apocynaceae | -- | -- | 3 | -- | -- | -- |
| Apocynaceae | Chloropetalum | *C. denticulatum* | 1 | Anemo | C | Yes |
| Aquifoliaceae | Ilex | *I. paraguariensis* | 2 | Zoo | S, T | Yes |
| Araceae | Anthurium | *A. scandens* | 2 | Zoo | H | Yes |
| Araceae | Philodendron | *Philodendron* sp. | 19 | Zoo | C | Yes |
| Araliaceae | -- | -- | 1 | -- | -- | -- |
| Araliaceae | Didymopanax | *D. angustissimus* | 1 | Anemo | T | Yes |
| Arecaceae | -- | -- | 1 | -- | -- | -- |
| Asteraceae | -- | -- | 28 | -- | -- | -- |
| Asteraceae | Austroeupatorium | *A. inulaefolium* | 1 | Anemo | S | No |
| Asteraceae | Baccharis | *B. calvescens* | 1 | Anemo | S | No |
| Asteraceae | Baccharis | *B. junciformis* | 1 | Anemo | H | No |
| Asteraceae | Baccharis | *Baccharis* sp. | 11 | Anemo | H | No |
| Asteraceae | Chrysolaena | *C. platensis* | 4 | Anemo | SS | No |
| Asteraceae | Conyza | *C. bonariensis* | 2 | Anemo | H | No |
| Asteraceae | Cyrtocymura | *C. scorpioides* | 2 | Anemo | SS | No |
| Asteraceae | Mikania | *M. micrantha* | 3 | Anemo | C | No |
| Asteraceae | Piptocarpha | *P. axillaris* | 3 | Anemo | T | No |
| Asteraceae | Solidago | *S. chilensis* | 15 | Anemo | SS | No |
| Asteraceae | Sphagneticola | *S. trilobata* | 3 | Zoo | H | No |
| Asteraceae | Vernonanthura | *V. puberula* | 1 | Anemo | S | No |
| Begoniaceae | Begonia | *B. magdalenensis* | 5 | Anemo | H | Yes |
| Bignoniaceae | -- | -- | 7 | -- | -- | -- |
| Bignoniaceae | Amphilophium | *A. crucigerum* | 2 | Anemo | C | Yes |
| Blechnaceae | Salpichlaena | *S. volubilis* | 1 | Anemo | C | Yes |
| Burseraceae | Protium | *P. widgrenii* | 8 | Zoo | T | Yes |
| Campanulaceae | Centropogon | *C. cornutus* | 1 | Zoo | S | Yes |
| Clethraceae | Clethra | *C. scabra* | 9 | Anemo | S, T | No |
| Cyatheaceae | Alsophila | *A. setosa* | 8 | Anemo | T | Yes |
| Cyatheaceae | Cyathea | *Cyathea* sp. | 33 | Anemo | TF | Yes |
| Cyperaceae | -- | -- | 1 | -- | -- | -- |
| Cyperaceae | Rhynchospora | *R. rugosa* | 7 | Anemo/Hydro | H | No |
| Erythropalaceae | Heisteria | *H. silvianii* | 1 | Zoo | T | Yes |
| Fabaceae | -- | -- | 1 | -- | -- | -- |
| Fabaceae | Centrolobium | *C. robustum* | 1 | Anemo | T | Yes |
| Fabaceae | Dalbergia | *Dalbergia* sp. | 1 | Anemo | T | No |
| Fabaceae | Ormosia | *O. minor* | 1 | Zoo | T | Yes |
| Humiriaceae | -- | *--* | 1 | -- | -- | -- |
| Humiriaceae | Vantanea | *V. compacta* | 4 | Zoo | T | Yes |
| Lauraceae | -- | -- | 1 | -- | -- | -- |
| Loganiaceae | Strychnos | *S. brasiliensis* | 1 | Zoo | T | Yes |
| Malvaceae | Triumfetta | *T. semitriloba* | 1 | Epizoo | SS | No |
| Marantaceae | Stromanthe | *S. tonckat* | 5 | Zoo | H | Yes |
| Melastomataceae | -- | -- | 36 | Zoo | -- | -- |
| Melastomataceae | Bertolonia | *B. acuminata* | 18 | Auto | H | Yes |
| Melastomataceae | Chaetogastra | *Chaetogastra* sp. | 1 | Zoo | H, S, T | Yes |
| Melastomataceae | Henriettea | *H. umbelluliflora* | 1 | Zoo | T | Yes |
| Melastomataceae | Miconia | *Miconia* sp. | 23 | Zoo | S, T | Yes |
| Melastomataceae | Miconia | *M. latecrenata* | 4 | Zoo | S, T | No |
| Melastomataceae | Miconia | *M. trianae* | 1 | Zoo | T | Yes |
| Melastomataceae | Pleroma | *Pleroma* sp. | 10 | Zoo | S, T | No |
| Meliaceae | -- | -- | 1 | -- | -- | -- |
| Menispermaceae | Cissampelos | *Cissampelos* sp. | 9 | Zoo | C | No |
| Menispermaceae | Cissampelos | *C. andromorpha* | 1 | Zoo | C | No |
| Menispermaceae | Cissampelos | *C. pareira* | 13 | Zoo | C | Yes |
| Moraceae | Ficus | *Ficus* sp. | 2 | Zoo | S, T | Yes |
| Myrtaceae | -- | *--* | 27 | -- | -- | -- |
| Myrtaceae | Campomanesia | *C. guaviroba* | 1 | Zoo | T | Yes |
| Myrtaceae | Campomanesia | *C. xanthocarpa* | 2 | Zoo | T | Yes |
| Myrtaceae | Eugenia | *E. punicifolia* | 2 | Zoo | SS | Yes |
| Myrtaceae | Myrcia | *Myrcia* sp*.* | 1 | Zoo | S, T | Yes |
| Myrtaceae | Myrcia | *M. multiflora* | 11 | Zoo | S, T | Yes |
| Myrtaceae | Myrcia | *M. splendens* | 3 | Zoo | Tree | Yes |
| Nyctaginaceae | Guapira | *G. opposite* | 2 | Zoo | S, T | Yes |
| Onagraceae | -- | -- | 1 | -- | -- | -- |
| Onagraceae | Ludwigia | *L. octovalvis* | 5 | Auto | SS | No |
| Passifloraceae | Passiflora | *Passiflora* sp. | 1 | Zoo | C | No |
| Piperaceae | Piper | *Piper* sp. | 4 | Zoo | S, T | Yes |
| Poaceae | Chusquea | *C. oxylepis* | 3 | Anemo | H, B | No |
| Poaceae | Paspalum | *P. regnellii* | 1 | Anemo | H, G | No |
| Poaceae | Rugoloa | *R. pilosa* | 5 | Anemo | H, G | Yes |
| Poaceae | Setaria | *Setaria* sp. | 1 | Anemo | H, G | No |
| Poaceae | Setaria | *S. sulcata* | 1 | Anemo | H, G | No |
| Primulaceae | -- | -- | 2 | Zoo | -- | -- |
| Primulaceae | Myrsine | *M. coriacea* | 5 | Zoo | S, T | No |
| Rosaceae | Rubus | *Rubus* sp. | 1 | Zoo | H, S | No |
| Rosaceae | Rubus | *R. rosifolius* | 2 | Zoo | SS | No |
| Rosaceae | Prunus | *P. myrtifolia* | 1 | Zoo | T | Yes |
| Rubiaceae | -- | -- | 14 | Zoo | -- | -- |
| Rubiaceae | Coussarea | *C. contracta* | 7 | Zoo | S, T | Yes |
| Rubiaceae | Faramea | *F. montevidensis* | 4 | Zoo | S, T | No |
| Rubiaceae | Psychotria | *P. suterella* | 2 | Zoo | S | Yes |
| Rubiaceae | Rudgea | *R. jasminoides* | 5 | Zoo | S, T | Yes |
| Salicaceae | Xylosma | *Xylosma* sp. | 2 | Zoo | S, T | No |
| Sapindaceae | Serjania | *S. communis* | 2 | Anemo | C | No |
| Sapotaceae | -- | -- | 5 | -- | -- | -- |
| Smilacaceae | Smilax | *S. spicata* | 1 | Zoo | C | Yes |
| Solanaceae | Athenaea | *A. fasciculata* | 2 | Zoo | S | No |
| Solanaceae | Solanum | *Solanum* sp. | 30 | Zoo | S, T, H, C | Yes |
| Symplocaceae | Symplocos | *Symplocos* sp*.* | 11 | Zoo | S, T | Yes |
| Symplocaceae | Symplocos | *S. estrellensis* | 1 | Zoo | S, T | Yes |
| Urticaceae | -- | -- | 2 | -- | -- | -- |
| Verbenaceae | Lantana | *Lantana* sp*.* | 1 | Zoo | H, S | No |

**Supporting Information - References**

Ait Baamrane, M. A., Shehzad, W., Ouhammou, A., Abbad, A., Naimi, M., Coissac, E., ... & Znari, M. (2012). Assessment of the food habits of the Moroccan dorcas gazelle in M'Sabih Talaa, west central Morocco, using the trn L approach. *PLoS One*, 7(4), e35643. <https://doi.org/10.1371/journal.pone.0035643>

Axtner, J., Crampton-Platt, A., Hörig, L. A., Mohamed, A., Xu, C. C., Yu, D. W., & Wilting, A. (2019). An efficient and robust laboratory workflow and tetrapod database for larger scale environmental DNA studies. *GigaScience*, 8(4), giz029. <https://doi.org/10.1093/gigascience/giz029>

Barreto, S. B., Lima, L. H. A., Berlinck, C. N., Gestich, C. C., Villela, P. M. S., Magioli, M., & Saranholi, B. H. (2025). DNA metabarcoding reveals the influence of crops on the diet of a large group-living Neotropical ungulate. *Conservation Genetics*, 1-16.<https://doi.org/10.1007/s10592-025-01724-z>

Catchen, J., Hohenlohe, P. A., Bassham, S., Amores, A., & Cresko, W. A. (2013). Stacks: An analysis tool set for population genomics. *Molecular Ecology*, 22(11), 3124–3140. <https://doi.org/10.1111/mec.12354>

Edgar, R. (2010). Usearch. Lawrence Berkeley National Lab. (LBNL)

Erickson, D. L., Reed, E., Ramachandran, P., Bourg, N. A., McShea, W. J., & Ottesen, A. (2017). Reconstructing a herbivore's diet using a novel rbcL DNA mini-barcode for plants. *AoB Plants*, 9(3), plx015.<https://doi.org/10.1093/aobpla/plx015>

Mallott, E. K., Garber, P. A., & Malhi, R. S. (2018). trnL outperforms rbcL as a DNA metabarcoding marker when compared with the observed plant component of the diet of wild white-faced capuchins (Cebus capucinus, Primates). *PloS one*, 13(6), e0199556. <https://doi.org/10.1371/journal.pone.0199556>

Martin, M. (2011). Cutadapt removes adapter sequences from high-throughput sequencing reads. *EMBnet. Journal*, 17(1), 10–12. <https://doi.org/10.14806/ej.17.1.200>

Newmaster, S. G., Fazekas, A. J., & Ragupathy, S. D. N. A. (2006). DNA barcoding in land plants: evaluation of rbcL in a multigene tiered approach. *Botany*, 84(3), 335-341.<https://doi.org/10.1139/b06-047>

Palmieri, L., Bozza, E., & Giongo, L. (2009). Soft fruit traceability in food matrices using real-time PCR. *Nutrients*, 1(2), 316-328. <https://doi.org/10.3390/nu1020316>

Saranholi, B. H., Rodriguez‐Castro, K. G., Carvalho, C. S., Chahad‐Ehlers, S., Gestich, C. C., Andrade, S. C., ... & Galetti Jr, P. M. (2023). Comparing iDNA from mosquitoes and flies to survey mammals in a semi‐controlled Neotropical area. *Molecular Ecology Resources*, 23(8), 1790-1799. <https://doi.org/10.1111/1755-0998.13851>

Saranholi, B. H., França, F. M., Vogler, A. P., Barlow, J., Vaz de Mello, F. Z., Maldaner, M. E., ... & Galetti Jr, P. M. (2024). Testing and optimizing metabarcoding of iDNA from dung beetles to sample mammals in the hyperdiverse Neotropics. *Molecular Ecology Resources*, 24(5), e13961. <https://doi.org/10.1111/1755-0998.13961>

Taberlet, P., Coissac, E., Pompanon, F., Gielly, L., Miquel, C., Valentini, A., ... & Willerslev, E. (2007). Power and limitations of the chloroplast trn L (UAA) intron for plant DNA barcoding. *Nucleic acids research*, 35(3), e14-e14. <https://doi.org/10.1093/nar/gkl938>

Zhang, J., Kobert, K., Flouri, T., & Stamatakis, A. (2014). PEAR: A fast and accurate Illumina paired-end reAd mergeR. *Bioinformatics*, 30(5), 614–620. <https://doi.org/10.1093/bioinformatics/btt593>
